# From correlation to causation: dissecting the detection methodologies of oral bacterial-fungal crosstalk in health and disease

**DOI:** 10.3389/fmicb.2026.1937704

**Published:** 2026-10-12

**Authors:** Yixin Zhang, Nanyan Bian, Zhuojun Dong, Biao Ren, Juan Li

**Affiliations:** 1 State Key Laboratory of Oral Diseases, West China School of Stomatology, National Clinical Research Center for Oral Diseases, Med-X Center for Manufacturing, Sichuan University, Chengdu, China; 2 Department of Orthodontics, Affiliated Stomatology Hospital of Southwest Medical University, Luzhou, Sichuan, China; 3 Department of Orthodontics, State Key Laboratory of Oral Diseases and National Clinical Research Center for Oral Diseases, West China Hospital of Stomatology, Sichuan University, Chengdu, China

**Keywords:** bacterial-fungal interactions, biofilm, cross-kingdom crosstalk, detection methodologies, multi-omics, oral microbiome, pathogenic causality

## Abstract

In the oral microecosystem, cross-kingdom interactions between bacteria and fungi are pivotal ecological events that determine the equilibrium between oral health and disease. Historically, technical limitations have confined research to describing correlations in microbial community composition, hindering the elucidation of underlying pathogenic mechanisms. This review aims to systematically outline the evolution of detection technologies for oral bacterial-fungal interactions and critically discuss the paradigm shift from identifying “associative correlations” to deciphering “causal mechanisms.” The literature search encompassed studies ranging from traditional culture and molecular identification to high-throughput sequencing for ecological network construction, as well as cutting-edge multi-omics integration and high-resolution imaging technologies. Beyond cataloguing technical advances, we critically evaluate the level of causal inference supported by each methodological class, with particular attention to temporality, confounding, ecological simplification, and the need for perturbational validation in disease-relevant models. Representative studies are used to illustrate how evidence can progress from co-occurrence and spatial proximity to functional dependency and mechanism.

## 1 Introduction

As the initial portal of the human digestive tract, the oral cavity harbors a highly complex and dynamic microbial ecosystem (Kitamoto et al., 2020; Sedghi et al., 2021). Research on the oral microbiome has traditionally focused on bacterial communities (Palmer Jr, 2014; Colombo and Tanner, 2019). However, advances in metagenomics, culturomics, and fungal profiling have increasingly highlighted the importance of the oral mycobiome in maintaining microbial homeostasis and driving disease progression (Liu et al., 2023). Fungi are integral components of the oral microbiome, but disease-associated shifts in fungal composition do not by themselves establish that fungi drive pathology. Experimental studies showing that *Candida albicans* can reshape bacterial community composition and promote cariogenic dysbiosis provide a mechanistic bridge between community change and disease (Ghannoum et al., 2010; Du et al., 2021a). Because dysbiosis may be both a cause and a consequence of disease, establishing temporal direction and excluding disease-driven ecological selection remain central challenges in causal studies.

Oral microorganisms rarely exist as isolated species but instead establish extensive cross-kingdom interactions. Through mechanisms including metabolic cross-feeding, quorum sensing, physical adhesion, and environmental modification, bacterial-fungal interactions influence microbial growth, biofilm architecture, ecological stability, and pathogenic potential (Sztajer et al., 2014; Du et al., 2022). These relationships span metabolic cooperation, competitive antagonism, and context-dependent commensalism, and their net effect can alter biofilm structure, resilience, and pathogenic potential. Their clinical relevance is therefore best considered at the level of interacting microbial consortia: a disease phenotype may arise from an interaction network even when no single organism is uniquely abundant or sufficient to explain the phenotype.

Although high-throughput sequencing has greatly improved the comprehensive profiling of microbial communities and enabled the identification of disease-associated bacterial-fungal co-occurrence patterns, correlation-based analyses remain inherently limited (Noecker et al., 2019; Ji et al., 2025). Characterizing microbial composition alone cannot explain microbial functions, interspecies communication, or the mechanisms through which microorganisms influence disease progression. Consequently, distinguishing causative interactions from ecological associations has become one of the central challenges in oral microbiome research (Gilbert et al., 2016; Gu et al., 2020). Here, correlation refers to statistical co-variation between measured microbial features, whereas association is used more broadly for a reproducible relationship that may still reflect confounding or shared environmental responses. We reserve causation for evidence that perturbing a microbial member or defined interaction produces a corresponding change in a biological or disease phenotype (Hill, 1965; Lv et al., 2021).

Increasing evidence indicates that bacterial-fungal interactions are mediated by specific molecular mechanisms rather than simple co-occurrence. These mechanisms include metabolic exchange, such as short-chain fatty acid (SCFA) metabolism, signaling molecule-mediated communication, extracellular matrix remodeling, and spatial organization within polymicrobial biofilms (Diaz et al., 2014; Lamont et al., 2018; Miller et al., 2019; Lapiere and Richard, 2022). Rather than functioning solely as independent pathogens, fungi can modify bacterial behavior and community function, thereby influencing biofilm maturation, antimicrobial tolerance, and host tissue responses (Jiang and Lin, 2026). Understanding these interactions is useful because it links ecological observations to specific pathophysiological processes: in caries, cross-kingdom cooperation can intensify acidogenic and matrix-forming phenotypes, whereas in periodontitis it may influence epithelial injury and inflammatory signaling. Resolving those links requires methods that integrate taxonomic, functional, metabolic, spatial, and perturbational evidence rather than relying on co-occurrence alone.

In this review, we systematically summarize the evolution of methodologies used to investigate oral bacterial-fungal interactions, spanning traditional isolation and culture techniques, molecular identification, high-throughput sequencing, multi-omics integration, advanced imaging technologies, and experimental models for causal validation. Particular emphasis is placed on how these methodological advances enable the dissection of metabolic interactions, signaling networks, spatial organization, and functional dependencies underlying cross-kingdom crosstalk. By highlighting both the strengths and limitations of current approaches, this review aims to provide a methodological framework for elucidating the ecological mechanisms governing oral health and disease. Representative oral bacterial and fungal taxa involved in cross-kingdom interactions are summarized in Figure 1. Accordingly, we distinguish methods that primarily identify associations from those that provide mechanistic or interventional evidence.

**Figure 1:**
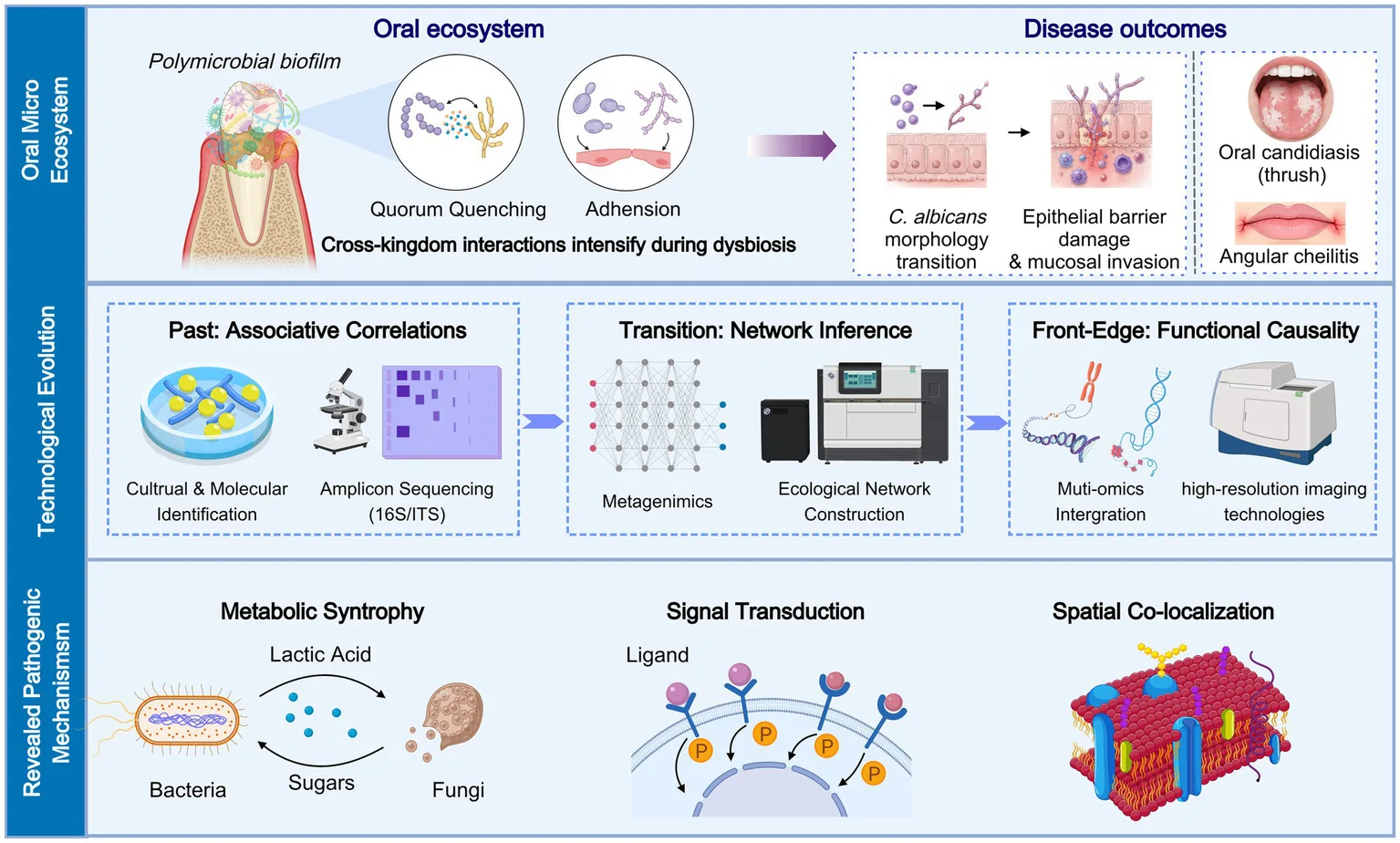
From association to causality: technological advances and pathogenic mechanisms in oral bacterial–fungal interactions. Schematic illustration of cross-kingdom interactions within the oral microbiome. Oral bacteria and fungi coexist in biofilms and engage in dynamic interactions through adhesion, metabolic exchange, and signaling communication. These interactions contribute to either microbial homeostasis or dysbiosis. Key bacterial and fungal taxa commonly detected in the oral cavity are summarized, highlighting their representative morphologies and ecological roles.

## 2 Historical perspective: from culture-dependent isolation to molecular identification

The foundation of traditional microbiological research lies in pure culture techniques, which have enabled the phenotypic and physiological characterization of individual microorganisms since the establishment of classical microbiology (Fredericks and Relman, 1996). Early investigations of oral bacterial-fungal interactions primarily relied on selective culture media to isolate clinically relevant microorganisms, such as *Candida albicans* and *Streptococcus mutans* (Frey-Klett et al., 2011). Pure culture remains indispensable for assigning intrinsic phenotypes to individual microorganisms, but its causal scope is limited because it removes the ecological context in which those phenotypes operate. In caries, for example, acid production by an isolated streptococcus demonstrates metabolic capacity rather than a sufficient causal contribution to lesion development, because saliva, dietary substrate, fungal partners, and other community members can substantially modify the phenotype. In addition, uncultivated or fastidious taxa are systematically underrepresented, so conclusions drawn from cultivable isolates may not capture the full set of interactions present in disease-associated consortia (He and Cheng, 2025; Zhang and Zhai, 2025; Dormiente et al., 2026).

To overcome these limitations, *in vitro* co-culture systems were developed to support the simultaneous growth of bacteria and fungi within a shared environment (Espinosa-Ortiz et al., 2022). These models represented the first experimental platform for investigating interspecies interactions and enabled causal perturbation experiments by selectively manipulating microbial partners. Co-culture studies revealed that bacterial metabolites can promote or inhibit fungal morphological transitions, including the yeast-to-hypha switch, thereby providing mechanistic insights into bacterial-fungal communication (Chen et al., 2020; Negrini et al., 2022; Gilbert Klaczko et al., 2023). Although co-culture simulates a multispecies coexistence environment to a certain extent, it is often limited to pairwise combinations of a few known species (Faust and Raes, 2012; Deng et al., 2019a). Nevertheless, most co-culture models remain restricted to pairwise combinations of predefined species and fail to recapitulate the ecological complexity, nutritional gradients, and signaling networks characteristic of the oral microbiome (Li et al., 2017; Zhou et al., 2022; Lueyar et al., 2023). For dental caries, *S. mutans*-*C. albicans* co-culture can test direct synergy, but pairwise systems may overestimate effects that depend on diet, saliva, host factors, or additional community members *in vivo*.

These limitations stimulated the development of culture-independent molecular approaches capable of directly interrogating complex microbial communities. Polymerase chain reaction (PCR) and quantitative PCR (qPCR) enabled the sensitive detection and quantification of predefined bacterial and fungal taxa without requiring cultivation, thereby substantially expanding microbial surveillance in clinical samples such as saliva, supragingival plaque, and subgingival plaque (Yang et al., 2022). For example, species-specific qPCR assays have been used to quantify major cariogenic and periodontal bacteria, including *Streptococcus mutans*, *Streptococcus sobrinus*, *Aggregatibacter actinomycetemcomitans*, and *Porphyromonas gingivalis*, as well as *Candida albicans* in oral samples (Morillo et al., 2003; Yang et al., 2022). However, because these approaches depend on predefined primers and, in some assays, sequence-specific probes, they test a hypothesis that has already been narrowed to selected taxa or genes and provide little information about unmeasured partners, spatial organization, or direction of interaction (Bustin et al., 2009). In periodontitis, for example, simultaneous increases in *P. gingivalis* and Candida detected by qPCR would document co-enrichment but could still arise from a shared anaerobic or inflammatory niche rather than direct cross-kingdom cooperation. Absolute load, temporal sampling, and experimental perturbation are therefore needed before abundance changes can be interpreted causally.

Fluorescence *in situ* hybridization (FISH) further advanced the field by combining taxon-specific fluorescent probes with microscopic imaging, allowing direct visualization of bacterial and fungal spatial organization within intact biofilms (Pernthaler et al., 2001). Multiplex FISH analyses of natural dental plaque revealed intimate spatial associations between oral streptococci and *C. albicans* hyphae, including characteristic corn-cob-like bacterial arrangements surrounding fungal filaments (Zijnge et al., 2010). These observations provide strong evidence that candidate partners occupy the same microenvironment, but spatial proximity is a prerequisite for many interactions rather than proof that one organism changes the behavior of the other. In plaque from caries or periodontal sites, co-localization may reflect shared attachment preferences or common environmental selection without implying metabolic exchange or causal interdependence. FISH therefore strengthens biological plausibility most effectively when paired with functional readouts, isotope tracing, or perturbation of the proposed interaction, motivating the subsequent development of multi-omics and experimental validation approaches.

## 3 High-throughput sequencing: mapping ecological networks

### 3.1 16S rRNA gene sequencing: profiling bacterial communities

16S rRNA gene sequencing is currently one of the most established and widely used approaches for characterizing the structure of oral bacterial communities. By amplifying specific hypervariable regions of the bacterial 16S rRNA gene, such as the V3–V4 regions, followed by deep sequencing on high-throughput platforms, this technique provides comprehensive information on bacterial taxonomic composition, relative abundance, and community diversity. In studies of oral bacterial-fungal interactions, 16S rRNA gene sequencing can delineate changes in bacterial α-diversity, assessed using indices such as Shannon and Chao1, and β-diversity, commonly evaluated by principal coordinate analysis, under pathological conditions including dental caries, periodontitis, and oral lichen planus. For example, 16S rRNA gene sequencing has shown that children with severe early childhood caries exhibit marked bacterial dysbiosis, characterized by reduced microbial diversity and an increased relative abundance of cariogenic taxa such as *Streptococcus mutans*, *Bifidobacterium*, and *Scardovia* spp. (Hurley et al., 2019). More recent studies using this approach have further characterized dynamic changes in the oral microbiome across different health and disease states, including oral and gastric microbial profiles in patients with early-stage intramucosal esophageal squamous cell carcinoma, plaque microbiota across different caries-risk groups in preschool children (Jiang and Lin, 2026), and differences in supragingival microbial communities between caries-free and caries-active adolescents undergoing fixed orthodontic treatment (Miao et al., 2025). Such case–control profiles are valuable for identifying disease-associated candidates, but relative-abundance data are compositional and can change because one taxon expands, another contracts, or the total microbial load shifts (Gloor et al., 2017; Knight et al., 2018). In caries, apparent enrichment of a taxon may therefore reflect ecological selection by an already acidic environment rather than a role in disease initiation. Longitudinal sampling, absolute quantification, and targeted perturbation are needed to distinguish temporal precedence from secondary expansion.

### 3.2 ITS sequencing: challenges in fungal identification and the importance of reference databases

In contrast to bacterial profiling based on 16S rRNA gene sequencing, fungal community analysis primarily relies on amplicon sequencing of the internal transcribed spacer (ITS) regions. The ITS1 and ITS2 regions exhibit substantial sequence variability among fungal taxa and are widely regarded as standard DNA barcodes for fungal identification. However, profiling the oral mycobiome presents greater technical challenges than profiling the bacteriome. First, fungal biomass in oral samples is typically much lower than bacterial biomass, making fungal analyses particularly vulnerable to interference from abundant host DNA, environmental contamination, primer bias, and incomplete reference databases. Second, fungal taxonomic assignment depends heavily on the completeness and accuracy of available reference databases. Although commonly used databases such as UNITE are continuously updated, taxonomic annotations for some rare, poorly characterized, or uncultured oral fungal taxa remain limited. Therefore, high-quality ITS sequencing requires not only rigorous experimental procedures for fungal DNA enrichment and contamination control, but also the use of up-to-date reference databases and stringent bioinformatic pipelines to ensure accurate identification of common oral fungi, including *Candida* and *Saccharomyces*. These challenges are particularly relevant in studies examining how specific environmental exposures, such as heavy metals, influence fungal diversity and the network structure of the oral mycobiome (Li et al., 2025). From a causal perspective, these technical sources of bias are not merely analytical details: in low-biomass oral fungal communities, contamination or primer preference can create or obscure apparent disease associations. An increase in Candida reads in candidiasis, caries, or periodontitis should therefore be interpreted together with independent fungal-load measurements, clinical context, and functional evidence rather than as proof that the detected fungal shift initiated disease. Replication across extraction protocols or primer sets can strengthen robustness, but it does not substitute for temporal or experimental validation (Knight et al., 2018).

### 3.3 Co-occurrence network analysis

After bacterial and fungal community profiles have been generated using 16S rRNA gene and ITS sequencing, respectively, co-occurrence network analysis is commonly used to investigate potential cross-kingdom associations. This approach constructs ecological networks by estimating the strength of abundance-based associations among microbial operational taxonomic units (OTUs) or amplicon sequence variants (ASVs) across samples. Commonly used methods include SparCC (Sparse Correlations for Compositional Data) and SPIEC-EASI (Sparse Inverse Covariance Estimation for Ecological Association Inference). These algorithms are designed to account, under specific statistical assumptions, for the compositional nature of sequencing data and thereby improve the inference of positive associations, which may suggest synergistic or mutualistic relationships, and negative associations, which may reflect competition or mutual exclusion. For example, in subgingival plaque networks from patients with periodontitis, specific anaerobic bacteria have been reported to show significant positive associations with *Candida* species (Khan et al., 2024). Recent studies have further demonstrated the utility of network-based approaches for investigating oral microbial ecology, including periodontal disease-associated alterations in the functional potential and ecological network structure of the canine oral microbiota (Park et al., 2026), as well as bacterial-fungal associations in esophageal cancer and precancerous lesions (Yuan et al., 2025). Accordingly, a network edge should be interpreted as a conditional association rather than a direct biological interaction. In periodontitis, two taxa can become positively connected because both favor the same inflamed, anaerobic niche, while preprocessing choices and compositional constraints can alter the inferred network topology (Gloor et al., 2017; Hirano and Takemoto, 2019). Edges become more persuasive as interaction hypotheses when they recur across cohorts and are supported by spatial, metabolic, or perturbational evidence.

### 3.4 Limitations

Co-occurrence does not necessarily indicate a direct physical or metabolic interaction. Although network analysis provides a broad ecological overview of the oral microbiome, its inherent limitations must be carefully considered. Most importantly, network analysis identifies statistical associations rather than causal relationships. A strong association between two microbial taxa may arise because both respond to a shared environmental factor, such as pH, redox potential, nutrient availability, or host immune status, rather than because they interact directly. In addition, network analysis cannot reliably distinguish direct interactions, such as the exchange of signaling molecules or metabolites, from indirect interactions mediated through changes in the surrounding environment. Consequently, sequencing-based co-occurrence networks are generally regarded as hypothesis-generating tools rather than definitive evidence of biological interaction (Hirano and Takemoto, 2019). Predicted associations therefore require functional validation using complementary approaches, such as *in vitro* co-culture systems, metabolomic analyses, spatial imaging, or gnotobiotic animal models, before their biological significance and causal relevance can be established.

## 4 Bridging the gap: functional and mechanistic evidence

Transcriptomics, particularly RNA sequencing (RNA-seq), is a key approach for deciphering microbial functional activity and enables comprehensive characterization of gene expression profiles in multispecies biofilms under defined physiological or pathological conditions (He et al., 2017; Hu et al., 2024). This technology allows researchers not only to profile community-wide transcriptional activity but also, more importantly, to identify differentially expressed genes (DEGs) associated with interspecies signaling and interaction responses (Liu et al., 2022). For example, perturbation-based studies using bacterial-fungal co-culture systems have shown that the *S. mutans* SigX-inducing peptide suppresses *C. albicans* hyphal development, biofilm formation, and virulence through the Ras1–cAMP–Efg1 signaling pathway (Zhang et al., 2023). Nevertheless, differential expression identifies a response associated with co-culture and does not automatically identify the driver of that response. In *S. mutans*-*C. albicans* systems, transcriptional changes become stronger mechanistic evidence when the candidate bacterial signal, fungal regulator, or downstream pathway is genetically or pharmacologically perturbed and the interaction phenotype changes accordingly. Time-resolved expression can further distinguish an initiating response from transcriptional consequences that appear only after the biofilm phenotype has developed.

Proteomics and metabolomics provide complementary evidence at the protein and metabolite levels, respectively, and are therefore valuable for elucidating the functional mechanisms underlying microbial interactions (Xiong et al., 2022). Proteomics characterizes expressed proteins, secreted enzymes, and virulence factors, whereas metabolomics detects small-molecule mediators, including organic acids, quorum-sensing molecules, and metabolic cross-feeding products (Brennan et al., 2025). A representative example comes from metabolomic studies showing that lactic acid produced by oral streptococci can be efficiently utilized by *Candida* as a carbon source. Multi-omics analyses have further revealed coordinated carbohydrate metabolism and extensive metabolic cross-feeding within *S. mutans–C. albicans* biofilms, thereby influencing biofilm biomass and pathogenic potential (Ellepola et al., 2019). In cariogenic biofilms, metabolite or protein shifts can nominate exchanged substrates, virulence factors, and stress-response pathways, but abundance changes alone cannot determine whether they are causes or consequences of altered community structure. Causal attribution becomes stronger when the proposed producer, metabolite, enzyme, or pathway is selectively perturbed and the predicted biofilm phenotype changes, particularly if isotope tracing or rescue experiments confirm the direction of transfer. This distinction is important because the same metabolite may be a marker of a changed niche without being the mediator responsible for disease-related synergy.

Furthermore, shotgun metagenomic sequencing enables comprehensive assessment of the functional potential of the microbiome by sequencing all microbial genomic DNA present in a sample without targeted amplification. This approach overcomes the taxonomic limitations of 16S rRNA gene sequencing by linking shifts in microbial community composition to changes in the abundance of functional genes, including antibiotic resistance genes and genes involved in specific metabolic pathways. Metagenomic analysis therefore defines the functional potential encoded by a community rather than the activity of those functions at a specific time. In periodontitis or caries, enrichment of a virulence or metabolic gene does not establish that the gene is expressed, assigned to the relevant interacting strain, or required for the observed phenotype. Integrating metagenomics with metatranscriptomics, metabolomics, strain-resolved analysis, and targeted perturbation converts genomic potential into a more testable mechanistic hypothesis.

## 5 Spatial resolution: visualizing the interaction Interface

In oral microbial ecology, characterizing the spatial organization of microbial communities and their physical interfaces with host tissues is essential for understanding the pathogenic mechanisms and symbiotic relationships within biofilms. The integration of advanced microscopy with spatial multi-omics technologies allows these complex interactions to be visualized at the micro- and even nanoscale.

Advanced microscopy provides direct visual evidence of the three-dimensional architecture and ultrastructural features of oral biofilms. Confocal laser scanning microscopy (CLSM), combined with specific fluorescent probes or live/dead staining, can visualize the three-dimensional structure, biomass distribution, and co-aggregation of bacteria and fungi, such as *Candida albicans*, within multispecies biofilms formed on dentin surfaces or tissue-engineered materials (Ansbro et al., 2022). For example, in studies evaluating the activity of cinnamaldehyde against *Streptococcus mutans* biofilms, CLSM was used to demonstrate disruption of the three-dimensional biofilm architecture (He et al., 2019). CLSM is also widely used to assess the penetration of antimicrobial dentifrices or nanoparticles into biofilms. For causal interpretation, however, CLSM remains primarily a structural readout: increased co-aggregation or biomass does not reveal which partner initiated the change or whether the spatial arrangement is required for disease. In caries models, a denser *S. mutans*-*C. albicans* architecture becomes mechanistically informative when disruption of a specific adhesin, matrix component, or signaling pathway also reduces the relevant acidogenic or tissue-damaging phenotype. Quantitative image analysis should therefore be linked to defined perturbations rather than used as stand-alone evidence of synergy.

Electron microscopy provides substantially higher spatial resolution and enables detailed characterization of microbial–surface interfaces. Scanning electron microscopy (SEM) is commonly used to examine bacterial colonization on root surfaces, interactions among fimbriae and extracellular polymeric substances, and morphological damage following antimicrobial treatment. Transmission electron microscopy (TEM) can further reveal alterations in intracellular ultrastructure. For example, in studies evaluating EDTA and air-polishing powders for root-surface decontamination in periodontitis, SEM has been used to assess biofilm removal and dentinal tubule patency. Atomic force microscopy (AFM) has also been applied to compare the adhesion forces of probiotic and pathogenic microorganisms on dental surfaces.

To address the limited molecular specificity of conventional imaging methods, spatial multi-omics technologies have emerged, enabling a transition from morphological observation to spatially resolved molecular mapping. Imaging mass spectrometry allows label-free, *in situ* detection and spatial mapping of metabolites, lipids, and proteins across biofilm surfaces or tissue sections. This approach can reveal spatial gradients of metabolites, including organic acids and signaling molecules, around different microbial populations within oral biofilms, thereby providing insight into interspecies competition and metabolic cross-feeding. For example, imaging-based metabolomic approaches can be used to characterize the spatial metabolite profiles of subgingival plaque from patients with periodontitis (Ashfaq et al., 2025).

Fluorescence *in situ* hybridization coupled with secondary ion mass spectrometry (FISH-SIMS) further links microbial identity with metabolic activity at the single-cell level. By combining taxon-specific fluorescent labeling with the isotope-detection capability of SIMS, this approach can localize specific microorganisms, such as *Porphyromonas gingivalis*, within complex communities while simultaneously measuring the incorporation of isotope-labeled substrates. It therefore enables quantitative assessment of microbial metabolic activity and nutrient uptake within defined microenvironments (Behrens et al., 2008).

## 6 Application of fungal-bacterial interaction models in investigating mechanisms and causal relationships

In oral microbiome research, the transition from correlation to causal validation relies heavily on complementary *in vitro* and *in vivo* models. *In vitro* systems provide controlled and reproducible platforms for dissecting the mechanisms underlying bacterial–fungal cross-kingdom interactions while approximating key features of the oral microenvironment. Among these, flow-cell systems operated under salivary flow conditions have been used to investigate bacterial–fungal adhesion, spatial organization, and biofilm development under conditions that more closely resemble the oral cavity (Diaz et al., 2012). Compared with static cultures, flow-cell and multispecies systems improve ecological realism by introducing shear, nutrient delivery, and community competition, and they permit controlled perturbation of specific partners (Bravo et al., 2024; Cao et al., 2025). Their strongest causal contribution comes from showing that removing or altering a candidate interaction changes a prespecified functional phenotype under otherwise matched conditions. Even then, the absence of immune, epithelial, dietary, or salivary components limits direct extrapolation from biofilm causality to clinical disease causality.

The incorporation of three-dimensional organoids, reconstructed human oral epithelium (RHOE) models, such as EpiOral™, and *in vivo* animal models further enables validation of the pathogenic mechanisms of cross-kingdom interactions in more complex biological systems. By introducing a host epithelial barrier, 3D organoids and EpiOral™ models allow direct assessment of how bacterial–fungal co-aggregation affects epithelial integrity and immune responses (Wronowska et al., 2025). Germ-free or gnotobiotic mice and oral infection models, in turn, can recapitulate disease progression within a living host (Du et al., 2021b). For example, models based on colonization with defined bacterial–fungal consortia, or on ligature-induced anaerobic conditions that favor the enrichment of cross-kingdom pathogens, can be used to validate *in vivo* the synergistic tissue-destructive effects of virulence mechanisms identified *in vitro* (Table 1) (Veerapandian et al., 2024; Abe et al., 2026). This integrated framework is most persuasive when a mechanism identified *in vitro* is perturbed *in vivo* and produces a concordant change in a disease-relevant phenotype. Animal models can therefore strengthen evidence for necessity or sufficiency within a living host, but their interpretation remains context dependent: host species, immune status, inoculation dose, diet, anatomical site, and simplified consortia can all alter the magnitude of the phenotype. Concordance across organotypic systems, animal models, and appropriately stratified human observations is consequently more informative than reliance on a single model.

**Table 1:** Application of *In Vitro* and *In Vivo* models for investigating mechanisms and causal relationships in oral bacterial–fungal interactions.

Year	Detection method/model used	Main conclusion	Reference
2025	3D organoid/epithelial model: using EpiOral™ 3D reconstructed human oral epithelium model, combined with LDH release and cytokine detection.	Validated that mixed biofilms of *P. gingivalis* and *C. albicans* disrupt epithelial barrier integrity more than single species and induce a strong pro-inflammatory response (IL-6, IL-8).	Bras et al. (2025)
2024	*In vivo* animal model: utilizing an immunosuppressed mouse model, combined with histopathology and microbiome sequencing.	Confirmed that oral candidiasis causes bacterial microbiome dysbiosis and promotes infection dissemination. Colonization by *C. albicans* alters the oral microenvironment, providing an ecological niche for pathogens.	Veerapandian et al. (2024)
2025	Gene-knockout dual-species biofilm model using *Candida albicans* V-ATPase gene mutants, combined with FISH, SEM, CLSM, EPS analysis, transcriptomics, and an in vivo rat caries model.	V-ATPase-related genes in *C. albicans* regulate hyphal development, biofilm architecture, EPS production, and the cariogenicity of *C. albicans–Streptococcus mutans* biofilms, supporting a causal role of fungal virulence pathways in cross-kingdom cariogenic biofilms.	Jiang et al. (2025)
2026	Commensal-antagonism biofilm model co-culturing H₂O₂-producing *Streptococcus oralis* with *S. mutans* and *C. albicans* on saliva-coated hydroxyapatite discs, combined with biochemical assays and time-lapse confocal imaging.	H₂O₂-producing commensal streptococci disrupt *S. mutans–C. albicans* synergism by inhibiting EPS formation, hyphal transition, and bacterial–fungal colocalization, supporting a causal antagonistic mechanism in cross-kingdom biofilms.	Shao et al. (2026)
2023	Virulence factor validation model: validating using gene knockout strains (e.g., *Δals1*, *Δhwp1*) in *in vitro* biofilm and cell co-culture systems.	Confirmed that *C. albicans* adhesins (*Als1*, *Hwp1*) are key virulence factors mediating physical co-aggregation with *S. mutans.*	Martorano-Fernandes et al. (2023)
2023	XIP treatment, hyphal-transition and biofilm assays, gene-expression analysis, cAMP measurement, pathway-rescue experiments, and an oropharyngeal candidiasis model.	Discovered that *S. mutans* SigX-inducing peptide (XIP) inhibits the hyphal transition of *C. albicans*, revealing the “underlying mechanism” by which bacteria regulate fungal morphogenesis via secreted signaling molecules.	Zhang et al. (2023)

## 7 Discussion: from correlation and association to causation in Oral microbial consortia

The literature reviewed here indicates that the transition from correlation and association to causation is not a linear progression in which newer technologies automatically provide stronger evidence. Rather, each method addresses a different component of the causal question. Cross-sectional sequencing and co-occurrence networks identify reproducible associations and candidate partners; longitudinal sampling adds temporal ordering; spatial imaging asks whether organisms or metabolites are positioned to interact; transcriptomics, proteomics, and metabolomics identify functional responses; and perturbation-based cultures, genetic manipulation, rescue experiments, organotypic systems, and animal models test whether changing a candidate interaction changes a defined phenotype. Causal confidence therefore depends on convergence across complementary approaches with different sources of bias rather than on any single platform (Hill, 1965; Fritz et al., 2013; Lv et al., 2021).

At the observational level, temporality and confounding remain major obstacles. Oral disease itself can alter pH, nutrient availability, oxygen tension, salivary composition, and host inflammatory responses, so two taxa may become correlated because they share the same altered niche rather than because they directly influence one another. This problem is particularly relevant to caries and periodontitis. For example, enrichment of cariogenic bacteria and Candida in an acidic plaque environment may reflect both ecological selection and biological cooperation, while positive bacterial-fungal network edges in periodontitis may arise partly from the inflamed, anaerobic subgingival environment. In addition, the compositional nature of sequencing data can generate apparent dependencies among relative abundances. Consequently, cross-sectional surveys and co-occurrence networks are most appropriately treated as hypothesis-generating evidence unless temporal, functional, or interventional data support a more direct interpretation (Gilbert et al., 2016; Hirano and Takemoto, 2019).

Spatial and multi-omics methods substantially strengthen biological plausibility, but they do not by themselves establish causation. FISH, CLSM, and related imaging approaches can demonstrate that bacterial and fungal cells occupy the same microenvironment, while transcriptomic, proteomic, and metabolomic analyses can identify coordinated pathway changes or candidate exchanged metabolites. In *S. mutans*-*C. albicans* biofilms, for instance, multi-omics data have revealed coordinated carbohydrate metabolism and metabolic cross-feeding (Ellepola et al., 2019). However, co-localization may be incidental, and a pathway or metabolite that changes during co-culture may be a downstream consequence rather than the driver of the phenotype. Stronger causal interpretation therefore requires perturbing the candidate microbial partner, signaling molecule, metabolite, receptor, or pathway and demonstrating a corresponding change in the interaction phenotype; rescue or complementation provides further support when feasible.

Perturbation experiments, gene knockout or rescue, and defined-consortium models provide stronger mechanistic evidence because they directly test whether a proposed interaction is necessary or sufficient for a phenotype. The *S. mutans* SigX-inducing peptide model illustrates this principle: manipulation of the bacterial signal together with pathway measurements links cross-kingdom communication to the *C. albicans* Ras1-cAMP-Efg1 pathway and virulence-related phenotypes (Zhang et al., 2023). Nevertheless, stronger internal validity is often obtained at the cost of ecological complexity. Pairwise cultures cannot fully reproduce the nutritional gradients, competitive interactions, salivary flow, host immunity, and community redundancy present *in vivo*. Three-dimensional epithelial models and animal studies restore part of this host context and can test whether a mechanism remains relevant to tissue damage or disease progression, but their conclusions are still influenced by host species, immune status, inoculation dose, and the composition of the experimental consortium (Falsetta et al., 2014; Du et al., 2021b; Wronowska et al., 2025).

These limitations are especially important when causal claims are translated across diseases. The same bacterial-fungal pair may have different effects depending on diet, oral hygiene, salivary flow, oxygen availability, host immunity, treatment exposure, and disease stage. Thus, a relationship that contributes to cariogenic biofilm acidification cannot automatically be assumed to have the same causal role in periodontitis, candidiasis, or other inflammatory oral conditions. Causality should therefore be considered at the level of specific functions and microbial consortia within a defined host and environmental context, rather than inferred from the abundance of a single taxon. Longitudinal human cohorts are particularly valuable for distinguishing interactions associated with disease initiation from those that emerge during progression or treatment, while careful stratification of disease stage and host exposures can reduce alternative explanations (Baas et al., 2024).

Taken together, these observations support a triangulation framework for causal inference in oral bacterial-fungal research. A practical sequence is to identify reproducible associations in well-characterized cohorts, test spatial and functional plausibility, perturb the proposed mechanism in controlled consortia, validate the resulting phenotype in organotypic or animal systems, and, where feasible, examine whether the same relationship predicts or responds to intervention in humans. Not every study must include every level of evidence, but causal language should be calibrated to the strength and consistency of the available evidence. Future work will benefit from predefined causal questions, explicit control of host and environmental confounders, time-resolved sampling, and replication across models with different assumptions. In this framework, the transition from correlation to causation is achieved through the accumulation and convergence of complementary evidence rather than through a single definitive technology.

## 8 Clinical implications of detecting cross-kingdom communication between oral bacteria and fungi

As mechanistic evidence for cross-kingdom interaction networks in the oral microbiome accumulates, clinical diagnostic and therapeutic strategies are shifting from the simple identification of pathogens toward the characterization of microbial interactions, thereby supporting the development of precision approaches based on ecological modulation (Chen et al., 2021; Mir, 2025). At the diagnostic level, the focus has expanded from detecting individual pathogens to identifying community-level dysbiosis and alterations in cross-kingdom metabolic networks. Cross-kingdom metabolites and signaling molecules are being investigated as candidate biomarkers of microbial dysbiosis, but a useful diagnostic marker must be reproducible across sampling conditions, disease stages, and relevant host subgroups and must add information beyond conventional clinical variables. Longitudinal cohorts are especially important because a marker that rises only after tissue damage has developed may be useful for classification without identifying an upstream causal process. Interaction-based signatures should therefore be evaluated separately for diagnostic performance, prognostic value, and mechanistic relevance before they are incorporated into risk prediction or early-intervention strategies.

At the therapeutic level, interventions targeting cross-kingdom interaction mechanisms are being explored as alternatives or adjuncts to conventional broad-spectrum antimicrobial strategies (Peng et al., 2022). Rather than indiscriminately eliminating microbial communities, these approaches aim to disrupt pathogenic interactions while restoring microbial homeostasis. Emerging therapeutic strategies therefore focus on key interaction nodes between bacteria and fungi (Deng et al., 2019b). Examples include inhibiting adhesion factors to prevent initial colonization and biofilm formation, applying quorum-quenching strategies to interfere with interspecies signaling and attenuate synergistic virulence, and introducing probiotics with cross-kingdom antagonistic activity to promote ecological replacement (Srivastava, 2017; Fleitas Martínez et al., 2019; Yin et al., 2022). For these approaches to be genuinely causality informed, the targeted interaction should first be shown to contribute reproducibly to the relevant disease phenotype rather than merely correlate with it. Therapeutic disruption must also be evaluated for ecological compensation, because suppressing one interaction node can open a niche for alternative pathogens or remove beneficial functions of commensal members. Most interaction-targeted strategies therefore remain preclinical, and efficacy against a defined interaction cannot be assumed to generalize across heterogeneous human disease states.

Future integration of longitudinal multi-omics data with machine-learning models may facilitate disease-risk stratification and prediction of treatment responses. However, individualized prediction based specifically on oral bacterial–fungal interaction networks remains at an investigational stage (Table 2) (Baimakhanova et al., 2025). Ultimately, targeting critical interaction nodes may help restore oral microbial homeostasis and limit disease progression. The clinical implications of detecting oral bacterial–fungal cross-kingdom communication, including interaction-based diagnostics and targeted ecological therapies, are illustrated in Figure 2. For caries and periodontitis, translation is further complicated by variation in diet, oral hygiene, host immunity, and disease stage.

**Table 2:** Summary of key studies and clinical implications of oral bacterial-fungal interactions.

Year	Detection method/Model used	Main conclusion	Reference
2014	In vivo animal model, confocal microscopy: utilizing a rat caries model, combined with fluorescent labeling to observe biofilm structure and determine acidogenic capacity and cariogenic phenotype.	*Streptococcus mutans* and *Candida albicans* form a symbiotic relationship in vivo; their synergistic interaction significantly enhances the virulence of plaque biofilms, leading to more severe dental caries.	Falsetta et al. (2014)
2015	Mutant screening, light microscopy, and fluorescence-based coaggregation assays were performed using a bacterial–fungal co-culture system to identify the cell-surface components that mediate interkingdom coaggregation.	Specific cell-surface components mediate coadhesion between *F. nucleatum* and *C. albicans*, potentially facilitating their colocalization in polymicrobial oral biofilms.	Wu et al. (2015)
2018	Gene knockout, in vitro co-culture: constructing *Streptococcus mutans* antigen I/II protein deletion mutants to observe changes in their in vitro co-aggregation ability with *Candida albicans.*	The *Streptococcus mutans* surface protein antigen I/II is a key factor mediating its interaction with *Candida albicans*; blocking this protein significantly reduces cross-kingdom biofilm formation.	Yang et al. (2018)
2020	Competitive inhibition assay, biofilm imaging: co-culturing commensal streptococci with cross-kingdom pathogens to analyze their inhibitory effects via microscopic imaging and biofilm quantification.	Certain commensal streptococci can effectively disrupt the synergy between *Streptococcus mutans* and *Candida albicans*, suggesting that microecological modulation (introducing beneficial bacteria) is a potential clinical strategy.	Huffines and Scoffield (2020)
2023	Enzyme activity assay, oxidative stress analysis: determining *Candida albicans* catalase activity to evaluate its protective effect on *Streptococcus mutans* in oxidative stress environments.	Catalase produced by *Candida albicans* protects *Streptococcus mutans* from oxidative stress damage; this catalase-mediated oxidative-stress cross-protection is an important mechanism of their cross-kingdom symbiosis.	Katrak et al. (2023)
2024	Microbiome sequencing: combining microbiome sequencing with metabolite analysis to explore influencing factors of cross-kingdom interactions in early childhood caries.	Cross-kingdom interaction plays a key role in early childhood caries and is affected by socioeconomic factors, suggesting that clinical prevention and control require comprehensive intervention combining ecological detection and patients’ living backgrounds.	Khan et al. (2024)
2025	Proteomics, ubiquitination modification analysis: utilizing mass spectrometry to analyze changes in *Candida albicans* protein modifications during cross-kingdom interactions.	*Streptococcus mutans* can regulate the ubiquitination modification process of *Candida albicans*; this alteration in post-translational modification affects fungal morphological transitions and virulence expression.	Zhang et al. (2025)
2025	In vitro antimicrobial assay, co-culture and mixed-biofilm assays, viable-cell enumeration, and atomic force microscopy to detect their inhibitory effects on various *Candida* species.	*Streptococcus dentisani* can effectively inhibit the growth of *Candida albicans* and *Candida glabrata*, suggesting that probiotic bacteria have potential supports further evaluation of *S. dentisani* as a potential probiotic candidate in preventing oral fungal infections.	Medina et al. (2025)
2025	V-ATPase-related gene-expression analysis, *VMA3/VMA4/VMA11* deletion mutants, growth and hyphal assays, dual-species biofilm analysis, and an in vivo caries model.	V-ATPase plays a key role in the acid tolerance and cariogenesis of cross-kingdom biofilms; V-ATPase-related pathways contribute to environmental adaptation, biofilm formation, and cariogenicity and may represent potential intervention targets.	Jiang et al. (2025)
2026	Hydrogen peroxide quantification, biofilm imaging: analyzing the capacity of oral commensal streptococci to produce hydrogen peroxide and its impact on cross-kingdom biofilm structure.	Commensal streptococci producing hydrogen peroxide can disrupt the synergy between *Streptococcus mutans* and *Candida albicans*; this natural chemical antagonistic mechanism could be exploited to provide a mechanistic basis for future ecological intervention strategies.	Shao et al. (2026)
2014	In vivo animal model, confocal microscopy: utilizing a rat caries model, combined with fluorescent labeling to observe biofilm structure and determine acidogenic capacity and cariogenic phenotype.	*Streptococcus mutans* and *Candida albicans* form a symbiotic relationship in vivo; their synergistic interaction significantly enhances the virulence of plaque biofilms, leading to more severe dental caries.	Falsetta et al. (2014)

**Figure 2:**
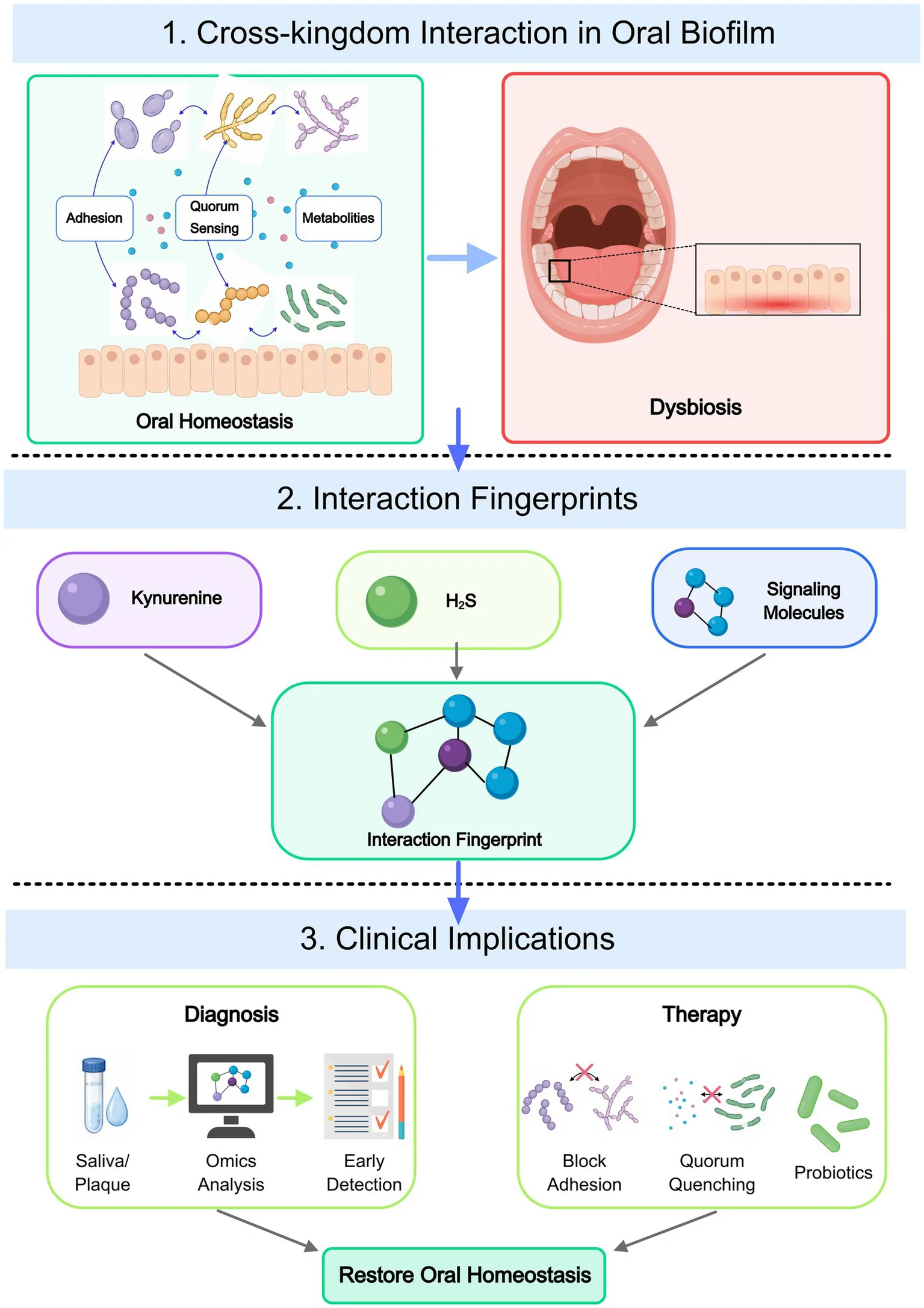
Clinical implications of detecting cross-kingdom bacterial–fungal interactions in the oral microbiome. Schematic representation of the clinical translation of oral bacterial–fungal interaction networks. Decoding cross-kingdom communication enables a shift from pathogen-centered diagnostics to interaction-based precision medicine. Metabolic and signaling interaction fingerprints may support future biomarker development, while targeted ecological interventions disrupt pathogenic bacterial–fungal alliances and restore microbial homeostasis.

## 9 Conclusion and future perspectives

We must emphasize that the synergistic pathogenicity between bacteria and fungi can contribute to the onset and progression of several oral diseases. Taking the “pathogenic alliance” formed by *Streptococcus mutans* and *Candida albicans* as an example, this cross-kingdom biofilm not only greatly enhances the mechanical stability of the microbial community through the physical “hyphal bridging” effect, but also significantly boosts the community’s drug resistance and environmental adaptability through metabolic syntrophy and co-regulation of gene expression (such as the upregulation of EPS synthesis) (Hwang et al., 2017; Xiang et al., 2025). Such synergistic pathogenicity may help explain why antibacterial treatment alone often fails to eradicate complex polymicrobial oral infections (Hu et al., 2021). Concurrently, the ecosystem also harbors natural antagonistic mechanisms. For instance, oral commensals (such as certain *Streptococcus* species) inhibit excessive fungal proliferation by secreting hydrogen peroxide or bacteriocins, while specific environmental factors (such as pH fluctuations and oxidative stress) interfere with cross-kingdom signaling molecules (e.g., farnesol) (Baty et al., 2022; Li et al., 2023). These mechanisms constitute an intrinsic defense line for maintaining microecological homeostasis. Unraveling the molecular details of these interactions marks a paradigm shift in our understanding of oral diseases, moving from the “single pathogen hypothesis” to the “ecological network pathogenic model” (Sedghi et al., 2021).

Future research should address several methodological and translational limitations in the study of oral bacterial–fungal interactions. To strengthen disease-specific causal interpretation, three priorities warrant particular attention.

First, basic research should prioritize longitudinal and time-resolved designs that establish whether a candidate interaction precedes, accompanies, or follows the disease phenotype. Because many studies still rely on *in vitro* co-cultures or cross-sectional omics analyses (Fritz et al., 2013; Baas et al., 2024), future work should combine repeated sampling with multispecies biofilms, organ-on-a-chip systems, and explicit control of diet, pH, salivary conditions, and host inflammatory state. This design strategy can separate interaction-specific effects from environmental changes that simultaneously influence several members of the consortium. By leveraging spatial transcriptomics, single-cell metabolomics, and isotope labeling techniques, we should strive to dissect the dynamic temporal characteristics of cross-kingdom interactions at single-cell resolution (Fritz et al., 2013; Baas et al., 2024). These approaches should generate spatially and temporally resolved maps of interspecies signaling and metabolic exchange and can be paired with targeted perturbation to test whether the mapped gradients are functionally required for the observed phenotype.

Second, clinical translation should first establish whether candidate cross-kingdom interactions are causal and reproducible in the relevant disease population. Anti-virulence and microbiome-modulating strategies can then be evaluated as targeted interventions. For example, this includes developing inhibitors that block the binding of bacterial-fungal adhesion receptors, or utilizing synthetic biology to design “smart probiotics” capable of specifically sensing and interfering with the formation of pathogenic cross-kingdom biofilms (Van Dyck et al., 2021). Furthermore, non-invasive biomarker strategies based on cross-kingdom co-occurrence patterns or metabolic fingerprints may ultimately support risk stratification in conditions such as oral cancer or severe periodontitis, but such signatures require prospective validation, disease-specific calibration, and evidence that they are not simply surrogates for inflammation or treatment exposure (Pös et al., 2023).

Third, regarding technological integration, the introduction of artificial intelligence and big data will accelerate breakthroughs in this field. By establishing databases of oral microbial cross-kingdom interactions and combining them with machine learning algorithms, we can predict the interaction outcomes (synergistic, antagonistic, or neutral) of different microbial combinations and screen for potential (Chen et al., 2025). This integrated computational–experimental framework (integrating computational and experimental approaches) will significantly reduce the cost of trial-and-error experimentation and enhance the efficiency of new drug development and the design of diagnostic and therapeutic protocols.

In summary, a mechanistic understanding of bacterial–fungal interactions may extend current ecological models of the oral microbiome and inform the development of interaction-based diagnostic and therapeutic strategies. We are shifting from simply “conquering microbes” to an ecological medicine paradigm of “living in harmony with microbes.” This transformation may not only provide evidence relevant to the future development of diagnostic and therapeutic strategies, but its research paradigm will also provide valuable reference for the study of other human microecosystems. However, clinical translation will require standardized analytical pipelines, longitudinal cohort studies, mechanistic validation, and controlled intervention trials. The central challenge is therefore not merely to detect cross-kingdom crosstalk, but to determine when an observed relationship is causal, disease-specific, and clinically relevant.
